# Common intensive care scoring systems do not outperform age and glasgow coma scale score in predicting mid-term mortality in patients with spontaneous intracerebral hemorrhage treated in the intensive care unit

**DOI:** 10.1186/s13049-017-0448-z

**Published:** 2017-10-25

**Authors:** Marika Fallenius, Markus B. Skrifvars, Matti Reinikainen, Stepani Bendel, Rahul Raj

**Affiliations:** 1 Division of Intensive Care, Department of Anesthesiology, Intensive Care and Pain Medicine, University of Helsinki and Helsinki University Hospital, Helsinki, Finland; 2 Australian and New Zealand Intensive Care Research Centre, School of Public Health and Preventive Medicine, Monash University, Melbourne, Australia; 3 Department of Intensive Care, North Karelia Central Hospital, Joensuu, Finland; 4 Department of Anesthesiology and Intensive Care, Kuopio University Hospital, Kuopio, Finland; 5 Department of Neurosurgery, University of Helsinki and Helsinki University Hospital, Helsinki, Finland

**Keywords:** Intracerebral hemorrhage, Hemorrhagic stroke, Intensive care, Outcome, Prognosis, Apache ii, Saps ii, Sofa, Glasgow coma scale score, Age

## Abstract

**Background:**

Intensive care scoring systems are widely used in intensive care units (ICU) around the world for case-mix adjustment in research and benchmarking. The aim of our study was to investigate the usefulness of common intensive care scoring systems in predicting mid-term mortality in patients with spontaneous intracerebral hemorrhage (ICH) treated in intensive care units (ICU).

**Methods:**

We performed a retrospective observational study including adult patients with spontaneous ICH treated in Finnish ICUs during 2003–2012. We used six-month mortality as the primary outcome of interest. We used logistic regression to customize Acute Physiology and Chronic Health Evaluation (APACHE) II, Simplified Acute Physiology Score (SAPS) II and Sequential Organ Failure Assessment (SOFA) for six-month mortality prediction. To assess the usefulness of the scoring systems, we compared their discrimination and calibration with two simpler models consisting of age, Glasgow Coma Scale (GCS) score, and premorbid functional status.

**Results:**

Totally 3218 patients were included. Overall six-month mortality was 48%. APACHE II and SAPS II outperformed SOFA (area under the receiver operator curve [AUC] 0.83 and 0.84, respectively, vs. 0.73) but did not show any benefit over the simpler models in terms of discrimination (AUC 0.84, *p* > 0.05 for all models). SAPS II showed satisfactory calibration (*p* = 0.058 in the Hosmer-Lemeshow test), whereas all other models showed poor calibration (*p* < 0.05).

**Discussion:**

In this retrospective multi-center study, we found that SAPS II and APACHE II were of no additional prognostic value to a simple model based on only age and GCS score for patients with ICH treated in the ICU. In fact, the major predictive ability of APACHE II and SAPS II comes from their age and GCS score components. SOFA performed significantly poorer than the other models and is not applicable as a prognostic model for ICH patients. All models displayed poor calibration, highlighting the need for improved prognostic models for ICH patients.

**Conclusion:**

The common intensive care scoring systems did not outperform a simpler model based on only age and GCS score. Thus, the use of previous intensive care scoring systems is not warranted in ICH patients.

## Background

The mortality of patients with spontaneous intracerebral hemorrhage (ICH) is markedly high. Studies from the U.S. and Europe have shown mortality rates as high as 30–60% one-year after the ICH [1–3]. Notably, is that mortality after ICH has not decreased recently [4, 5]. The existing guidelines for the management of spontaneous ICH [6] recommend blood pressure control, reversal of anticoagulation, glucose management, seizure treatment and selective surgery. The guidelines recommend initial treatment and monitoring in an intensive care unit (ICU) or a dedicated stroke unit [6]. Intensive care is, however, resource-demanding, and many patients face poor outcome. Prognostic models that provide prognostic information may aid in recourse allocation, improve ICH research by providing baseline risk stratification and improve comparison of cohorts in comparative effectiveness research [7]. Although common intensive care severity scores such as the Acute Physiology and Chronic Health Evaluation (APACHE) II [8] and Simplified Acute Physiology Score (SAPS) II [9] are commonly used for ICU-treated patients, they are both complex and non-specific for ICH patients. Furthermore, although not originally developed as a prediction model, the Sequential Organ Failure Assessment (SOFA) has also been used to predict outcomes of mixed-ICU populations, but has not specifically been tested in ICH patients [10, 11]. Earlier studies have suggested that both age and level of consciousness are some of the most important prognostic factors in patients with spontaneous ICH [12, 13]. Only a few small studies have, however, compared these single prognostic factors with more complex prognostic models in ICH outcome prediction.

We conducted this study to investigate the usefulness of common intensive care severity scores (APACHE II, SAPS II and SOFA) in predicting six-month mortality in patients with ICH treated in ICUs. We also investigated whether these scores are of any additional prognostic value compared to the value of simpler models. Based on our earlier study on traumatic brain injury (TBI) [14] we hypothesized that a simple model comprising age, premorbid functional ability, and level of consciousness performs as well as do more complex ICU scoring systems in predicting six-month mortality.

## Methods

### Study population and data collection

We conducted a retrospective observational study using a nationwide multi-center high quality ICU database, the Finnish Intensive Care Consortium (FICC). Ethical approval to conduct the study was obtained from the ethics committee of North Savonia hospital district (Dnro 30.03.2012 §19). The FICC database has been described in detail elsewhere [15, 16]. In short, the FICC database was established in 1994 to improve the quality of intensive care in Finland. It includes prospectively collected data on mortality and factors that affect prognosis such as co-morbidities and severity of illness from 21 different hospitals all over Finland. Data on physiologic variables is collected and validated from electronic patient monitoring systems and stored automatically. Data on co-morbidities, admission type, discharge status, and diagnoses are entered manually by ICU staff into the electronic database. The database is maintained by Tieto Healthcare & Welfare Ltd. (Helsinki, Finland).

**Fig. 1:**
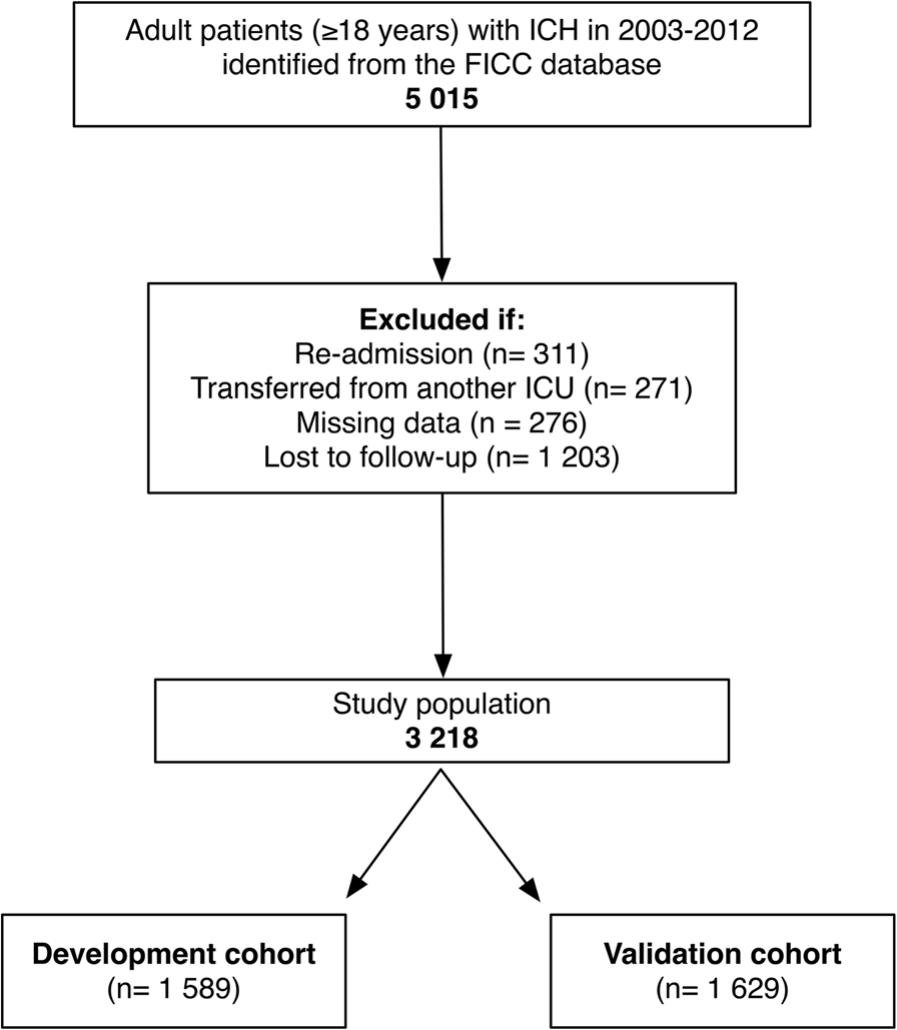
Study population. Abbreviations: FICC, Finnish Intensive Care Consortium; ICH, intracerebral hemorrhage; ICU, intensive care unit

From the database, we collected the APACHE II, SAPS II and SOFA scores. APACHE II and SAPS II are per definition collected during the first 24 h of ICU admission. For comparability, we used SOFA from the first ICU day [17]. We included adult patients (>18 years) treated for spontaneous ICH in Finnish ICUs between 2003 and 2012. We excluded patients being re-admitted or transferred from another ICU. Furthermore, patients with incomplete data, deficient APACHE II, SAPS II or SOFA data or those who were lost to follow-up were excluded (Fig. 1).

### Statistical analysis

For the statistical analyses, we used SPSS software for Macintosh, Version 23.0 (Armonk, NY: IBM Corp) and R: A Language Environment for Statistical Computing (R-Foundation for Statistical Computing, Vienna, Austria). We explored differences in baseline characteristics using a χ^2^ test (two-tailed) for categorical data and the Mann-Whitney U-test for continuous skewed data. The categorical data are presented as proportions (%) and continuous data as medians with inter-quartile ranges (IQR). We defined *p* values <0.05 as statistically significant.

To be able to provide a more accurate estimate of model performance, we internally validated all prognostic models [18]. In order to implement this, we applied a split-sample technique were the cohort was divided randomly into two independent parts named development and validation group [19].

All APACHE II, SAPS II, and SOFA severity scores use in-hospital mortality as endpoint. Yet many intensive care patients with poor prognosis are discharged to other institutions, where some will die during the following months. Therefore, using in-hospital mortality as the primary endpoint may bias the results [20]. Therefore, we wanted to investigate the adequacy of severity scores in predicting mid-term mortality and, thus, used six-month mortality as the primary outcome of interest. Hence, we used logistic regression analysis, using the logit-transformed original prediction as the independent variable and six-month mortality as the dependent variable, to first-level customize the ICU scores. First-level customization usually only improves calibration in the given data set without affecting discrimination [21]. We then created two different simpler reference models for comparison. The first one was composed of age and the worst GCS score measured during the first 24 h after ICU admission (reference-1). The second one included premorbid functional status in addition to age and GCS score (reference-2). Premorbid functional status describes the patient’s need for assistance in the daily life before hospitalization, and we categorized the patients into being either independent or dependent.

We evaluated the performance of the prognostic models by assessing discrimination and calibration. Discrimination is a measure of the model’s ability to distinguish between those patients who do experience the event of interest and those who do not. To measure discriminative ability, we constructed a receiver operator characteristic (ROC) curve and calculated the Area Under the Receiver Operator Curve (AUC). The AUC curve plots the combination of sensitivity and the complement of specificity covering the whole range of probabilities [22]. An AUC of >0.9 indicates excellent discrimination, 0.8–0.9 indicates good discrimination, 0.7–0.8 indicates satisfactory discrimination, 0.6–0.7 indicates poor discrimination, and an AUC of 0.5 indicates that the model does not predict better than mere chance.

Calibration is a measure of the model’s ability to generate estimates of risks that are in accordance with the observed outcomes at different classes of risk. To assess the models’ calibration, we used the Hosmer-Lemeshow (H-L) goodness-of-fit test. It examines how well the expected number of deaths are in accordance with the observed number of deaths over deciles of predicted risk. A *p*-value >0.05 indicates that the observed mortality does not differ significantly from the predicted and implies good calibration [23]. Although the H-L test is the most widely used test to measure calibration, it has been criticized for several reasons. First, H-L test plots average risk over deciles, not individual patients. Second, the calibration curve constructed to complement H-L test is not a curve, but a jagged line drawn between points [24]. Third, it has been criticized for being very sensitive to sample size [25]. For these reasons, we combined H-L goodness-of-fit test with a newer test for calibration, the GiViTI calibration belt [26, 27]. Unlike the calibration curve usually seen with the H-L test, the calibration belt offers both 80% confidence interval (CI; light gray area) and 95% CI (dark grey area) for the curve. The advantage of this new approach is that it allows to assess the degree of deviation from the ideal calibration line, and also to evaluate the direction of this phenomenon. When the 95% CI does not include the bisector line, the model is defined as poorly calibrated in that specific risk interval [24, 26].

### Post-hoc analyses

In response to a reviewer comment, we assessed the discrimination of the calibrated SAPS II and APACHE II scores without the age and GCS score components in a cohort including all patients (development and validation cohort).

## Results

### Baseline characteristics

A total of 3218 patients from 21 different hospitals met the inclusion criteria and were included in the study. After the random splitting was performed, 1589 (49%) patients were stratified to the development cohort and 1629 (51%) to the validation cohort (Fig. 1 *)*.

The patients’ baseline characteristics are shown in Table 1. Patient median age was 60 years (IQR, 52–69) and 91% (*n* = 2917/3218) of the patients were independent in activities of daily living prior to admission. The overall six-month mortality was 48% (*n* = 1527/3218). Of the non-survivors, 45% (*n* = 695/1527) died in the ICU, and 75% (*n* = 1139/1527) died before hospital discharge. There were no significant differences in age, prior functional ability, level of consciousness, and comorbidities between the development and validation cohorts.

**Table 1:** Patient baseline characteristics according to six-month outcome

	All patients (*n* = 3218)	Development (*n* = 1589)	Validation (*n* = 1629)	*P*-value*	Survivors (*n* = 1691)	Non-survivors (*n* = 1527)	*P*-value†
Age, in years							
< 45	416 (13)	216 (14)	200 (12)	0.392	263 (16)	153 (10)	<0.001
45–75	2467 (77)	1202 (75)	1265 (78)		1283 (76)	1184 (78)	
> 75	335 (10)	171 (11)	164 (10)		145 (8)	190 (12)	
Level of care hospital							
Secondary (Central)	866 (27)	435 (27)	431 (27)	0.557	295 (17)	571 (37)	<0.001
Tertiary (University)	2352 (73)	1154 (73)	1198 (73)		1396 (83)	956 (63)	
Prior functional ability							
Independent	2917 (91)	1435 (90)	1482 (91)	0.515	1578 (93)	1339 (88)	<0.001
Dependent	301 (9)	154 (10)	147 (9)		113 (7)	188 (12)	
GCS							
3 to 8	1999 (62)	992 (62)	1007 (62)	0.764	654 (39)	1345 (88)	<0.001
9 to 12	485 (15)	243 (15)	242 (15)		374 (22)	111 (7)	
13 to 15	734 (23)	354 (22)	380 (23)		663 (39)	71 (5)	
APACHE II	23 (16, 28)	23 (16, 28)	22 (16, 28)	0.311	17 (12, 23)	27 (23, 31)	<0.001
SAPS II	47 (30, 57)	47 (30, 58)	46 (30, 57)	0.466	33 (23, 46)	56 (48, 63)	<0.001
SOFA	7 (4, 9)	7 (4, 9)	7 (4, 9)	0.073	5 (3, 8)	8 (6, 10)	<0.001
TISS-76‡							
Total score	73 (44, 143)	74 (44, 145)	72 (44, 141)	0.605	80 (44, 163)	68 (44, 122)	<0.001
Average score	28 (23, 33)	28 (23, 33)	28 (22, 32)	0.055	26 (21, 31)	29 (24, 34)	<0.001
ICU admission characteristics							
Mechanical ventilation	2437 (76)	1213 (76)	1224 (75)	0.428	1052 (62)	1385 (91)	<0.001
Operative admission	1105 (34)	564 (36)	541 (33)	0.173	677 (40)	428 (28)	<0.001
Comorbidity	303 (9)	157 (10)	146 (9)	0.373	125 (7)	178 (12)	<0.001
Length of stay, in days							
ICU	2 (1, 4)	2 (1, 4)	2 (1,4)	0.875	2 (1, 5)	1 (1, 3)	<0.001
Hospital	5 (2, 12)	5 (2,12)	5 (2,12)	0.502	8 (4, 15)	3 (1, 7)	<0.001
Mortality							
ICU	695 (22)	345 (22)	350 (22)	0.876	NA	695 (46)	NA
Hospital	1139 (35)	559 (35)	580 (36)	0.801	NA	1139 (75)	NA
Six-month	1527 (48)	741 (47)	786 (48)	0.358	NA	1527 (100)	NA

Categorical variables are presented as n (%), all continuous variables were highly skewed and are therefore presented as median (IQR); *APACHE II* Acute Physiology And Chronic Health Evaluation II; *GCS* Glasgow Coma Scale; *ICU* intensive care unit; *NA* not available; *SAPS II* Simplified Acute Physiology Score II; *SOFA* Sequental Organ Failure Assessment; *TISS-76* Therapeutic Intervention Scoring System 76. *Between development and validation cohorts. †Between the survivors and non-survivors. **‡** TISS-76 score calculations were done once each calendar day. The total score refers to the sum of all TISS score calculations during the ICU stay. The average score refers to the mean daily score

The relationship between age and GCS score with six-month mortality is shown in Table 2. Mortality was significantly higher for patients with GCS scores between 3 to 8 compared to those with higher GCS scores. Furthermore, the mortality rate rose dramatically with age (Table 2). The effect of age on mortality was most notable in patients with GCS scores between 9 and 12, as the mortality was only 8% for patients aged <40, but as high as 43% for patients aged ≥80.

**Table 2:** The relationship between age and Glasgow Coma Scale with six-month mortality

Mortality, % (absolute numbers)				
Age, years	All patients (*n* = 3218)	GCS 3 to 8 (*n* = 1999)	GCS 9 to 12 (*n* = 485)	GCS 13 to15 (*n* = 734)
<40	34 (88/258)	57 (83/146)	8 (3/40)	3 (2/72)
40 to 49	41 (171/415)	66 (155/235)	11 (6/56)	8 (10/124)
50 to 59	47 (400/860)	65 (360/557)	21 (26/126)	8 (14/177)
60 to 69	47 (448/946)	67 (397/589)	21 (32/151)	9 (19/206)
70 to 79	58 (350/607)	75 (297/398)	39 (35/91)	15 (18/118)
≥ 80	53 (70/132)	71 (53/74)	43 (9/21)	22 (8/37)

### Six-month mortality prediction

Both APACHE II and SAPS II-based customized models showed good discrimination with AUCs of 0.83 (95% CI, 0.81–0.85) and 0.84 (95% CI, 0.82–0.86), respectively. Both reference models showed good discrimination with an AUC of 0.84 (95% CI, 0.82–0.86) for each. The reference models’ AUCs did not differ significantly from the AUCs of APACHE II and SAPS II (compared to APACHE II ΔAUC +0.01, p for refenrece-1 = 0.277, p for reference-2 = 0.336; compared to SAPS II, ΔAUC +0.00, p for reference-1 = 0.509, p for reference-2 = 0.466). The SOFA-based model showed significantly poorer performance compared to all other models, as its discrimination was only satisfactory with an AUC of 0.73 (95% CI, 0.71–0.76, compared to both reference models ΔAUC −0.11, *p* < 0.001) (Table 3).

**Table 3:** Scoring system performance for six-month mortality

Performance variable	Discrimination		Calibration	
	AUC	95% CI	H-L *P*-value	GiViTI *P*-value
Development cohort				
APACHE II	0.86	0.84, 0.88	0.766	NA
SAPS II	0.86	0.84, 0.88	0.624	NA
SOFA	0.74	0.71, 0.76	0.001	NA
Reference *	0.85	0.83, 0.86	0.001	NA
Reference †	0.85	0.83, 0.87	0.001	NA
Validation cohort				
APACHE II	0.83	0.81, 0.85	<0.001	<0.001
SAPS II	0.84	0.82, 0.86	0.058	0.014
SOFA	0.73	0.71, 0.76	<0.001	<0.001
Reference *	0.84	0.82, 0.86	<0.001	<0.001
Reference †	0.84	0.82, 0.86	<0.001	0.003

*APACHE II* Acute Physiology and Chronic Health Evaluation II; *GCS* Glasgow Coma Scale; *ICU* intensive care unit; *NA* not available; *SAPS II* Simplified Acute Physiology Score II; *SOFA* Sequential Organ Failure Assessment. *Reference model including age and GCS. † Reference model including age, GCS and premorbid functional status

**Fig. 2:**
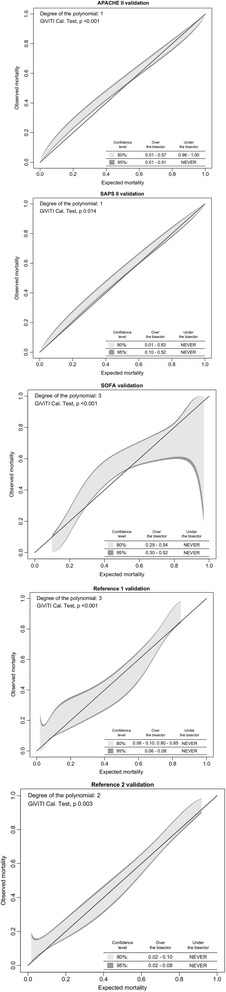
GiViTI calibration belt. Reference 1 refers to reference model including age and GCS. Reference 2 refers to reference model including age, GCS and premorbid functional status. All figures show a significant deviation from the bisector line indicating poor calibration. The deviation from ideal calibration is towards observed mortality, and therefore all models underestimated six-month mortality

The SAPS II-based model showed satisfactory calibration according to the H-L test with a *p*-value of 0.058. All other models showed poor calibration according to the H-L test with *P*-values <0.001. The GiViTI calibration belt showed poor calibration for all models as there were significant deviations from the bisector line for every model tested. The deviation from ideal calibration was towards observed mortality, and therefore all models underestimated six-month mortality (Fig. 2).

In the post-hoc analyses the calibrated SAPS II and APACHE II scores without their age and GCS score components had an AUC of 0.76 (95% CI 0.74–0.77) and 0.74 (0.72–0.75), respectively.

## Discussion

### Key findings

This is, by our knowledge, the largest study investigating the usefulness of common severity scores in predicting mid-term mortality in patients with spontaneous intracerebral hemorrhage treated in ICUs. Of the commonly used intensive care severity scores, both the APACHE II- and SAPS II-based models showed good discrimination, whereas SOFA displayed only satisfactory discrimination. In regard to calibration, only the SAPS II-based model showed satisfactory calibration whereas the other models showed poor calibration. In the post-hoc analyses, the discrimination of the SAPS II and APACHE II scores without their age and GCS score components markedly lowered their discriminative power. Thus, the main predictive ability of SAPS II and APACHE II in ICH patients comes from the strong predictive effect of age and the GCS score. This is strengthened by the study’s main finding, which is that compared to a simple prognostic model, including only age and GCS score, the more complex ICU scores were of no additional prognostic value. It is not surprising that SOFA did not match the predictive performances of APACHE II and SAPS II (or the simple age and GCS score model) as SOFA was originally intended as a descriptive measure of organ failure and not as a predictive measure. Thus, for ICH patients treated in the ICU, there is nothing to favor the use of previous complex ICU scoring systems, as age and GCS alone adequately predict mortality. Furthermore, abstracting age and GCS score is much more time-efficient than abstracting the complex intensive care scoring systems.

Interestingly, adding pre-admission functional status to the reference model (including age and GCS) did not improve the prognostic performance. This is somewhat surprising, as a recent study showed pre-admission functional status to be a strong independent predictor of outcome in general ICU patients [28]. Our results might indicate that in ICH patients, the injury severity itself is more important in determining patient prognosis than pre-admission functional status. Yet, only 9% of included patients were dependent in daily functions prior to admission. Thus, the effect of this variable is probably underpowered, which probably explains why it did not add any predictive power. Furthermore, included patients that were dependent prior to admission probably represent a selected cohort that have been considered to have a reasonable prognosis and therefore admitted to the ICU, increasing the likelihood of a type II error. Thus, any foregone conclusions regarding the association between pre-admission functional status and outcome cannot be drawn from our study.

### Comparison with previous studies

Clinical studies concerning the common intensive care severity scores in outcome prediction after ICH are limited, especially with regards to mid- or long-term mortality prediction. The results of our study are in concordance with previous studies. In a prospective study including 90 patients with acute stroke, *Handshu* et al. showed that the prognostic performance of GCS was almost equal to SAPS II in both 90-day (AUC 0.68, AUC 0.75 respectively) and 365-day mortality prediction (AUC 0.73, AUC 0.77 respectively) [13]. However, the study included both hemorrhagic (54%, *n* = 49) and ischemic stroke (46%, *n* = 41) patients and, thus, the results may be biased, as these are two very different patient populations. *Huang KB* et al. showed in a retrospective single-center study, including 75 patients, that APACHE II, SAPS II and ICH score predicted 30-day mortality well in patients with primary pontine hemorrhage (AUC for APACHE II 0.92, AUC for SAPS II 0.89, AUC for ICH score 0.84) [29]. Yet similarly to our study, the discriminative power of the GCS score (AUC 0.88) did not differ substantially from these more complex scoring systems. Furthermore, as in our study, SAPS II displayed the best calibration (*p* = 0.682). Patients with primary brain stem hemorrhage are, however, a specific group of stroke patients as their prognosis is significantly worse to other ICH patients. Additionally, in a large prospective study investigating the role of APACHE II in prediction of outcome after acute intracerebral hemorrhage, *Huang Y* et al. found the mortality prediction of APACHE II to correlate well with the observed outcome (*r* = 0.84, *p* < 0.001) [30]. The primary endpoint used was 3-month mortality, while we used six-month mortality as the primary outcome.

In this study, SOFA showed significantly poorer performance compared to the other models. This can be explained by the nature of the score itself. First, SOFA is an organ dysfunction score, originally designed to detect the degree of organ dysfunction instead of predicting outcome in critically ill patients. Second, the score is constructed of the level of dysfunction of six organ systems (cardiovascular, respiratory, hepatic, renal, coagulation, central nervous system) aiming to describe the degree of multi-organ failure which is common in sepsis, whereas ICH is more of a single organ problem, although multi-organ failure may occur [31]. In a large retrospective study investigating causes of death after ICH, *Zurasky* et al. found that only 9% of the deaths were due to non-neurologic reasons whereas neurological condition was the cause of death in overwhelming majority [32]. Also, SOFA does not consider patient age, which is a major prognostic factor in ICH patients.

Mortality in our sample is in line with previous studies, the six-month mortality being 48%. *Huang KB* et al. reported a 30-day mortality of 41% [29], whereas the three-month mortality was 40% in the study conducted by *Huang Y* et al. [30]. However, the mortality in the study conducted by *Handshu* et al. was substantially higher compared to all others, as the 90-day mortality was as high as 59% and one-year mortality being 68% [13].

In summary, the discriminative performance of a simple prognostic model composed of only age and GCS was equivalent to that of the more complex intensive care severity scores in patients with spontaneous ICH treated in the ICU. Thus, in regard to discriminative power, the age and GCS score based model can replace the previous severity scores. Yet, all models showed relatively poor calibration in predicting six-month mortality. Thus, as the clinical utility of a predictive model is influenced by both its discrimination and calibration [33] additional studies are necessary to improve the quality of predictive models used for quality assurance and research in intensive care for patients with spontaneous ICH. Furthermore, future studies should also take into account radiological parameters of the ICHs to improve the prognostic accuracy.

### Strengths and limitations

The major strength of our study is its adequate power to detect an effect, as our sample size is large, consisting of 3218 patients and up to our knowledge the largest study of this type published so far. Also, the majority of all ICUs within one country were involved, which improves generalizability. An additional strength of the study is the high quality of the database used [16]. There are, however, some limitations to this study that deserve attention. First, as the study is retrospective in nature we are restricted to the data available in the database. The FICC database is not a specific neurological ICU-database and it does not include variables that may be of specific interest in ICH patients, such as radiological data or information regarding use of anticoagulation medication. Thus, we were unable to get data on measures of ICH radiological parameters such as hematoma volume, intraventricular hemorrhage, and ICH location. Therefore, we are unable to study the performance of radiological scores, such as the ICH score, which has proved useful [34]. Second, as the management practices differ and ICU admission criteria are not equal, our findings may not be generally applicable to different healthcare systems in all cases.

## Conclusion

APACHE II and SAPS II showed good discrimination, while SOFA only satisfactory discrimination for predicting six-month mortality in in ICH patients. Only the SAPS II-based prediction model showed satisfactory calibration, whereas the other models displayed poor calibration. The APACHE II and SAPS II scoring systems did not outperform a simpler model based on only age and GCS score. Thus, the use of previous common intensive care scoring systems is not warranted in ICH patients.

## Abbreviations

APACHE II: Acute Physiology and Chronic Health Evaluation II
AUC: area under the receiver operator curve
CI: confidence interval
FICC: Finnish Intensive Care Consortium
GCS: Glasgow Coma Scale
GiViTI: Italian Group for the Evaluation of Intervention in Intensive Care Medicine
H-L test: Hosmer-Lemeshow goodness-of-fit test
ICH: intracerebral hemorrhage
ICU: intensive care unit
IQR: inter-quartile range
ROC: receiver operator characteristic curve
SAPS II: Simplified Acute Physiology Score II
SOFA: Sequential Organ Failure Assessment
TBI: traumatic brain injury
